# Supplementary material for: Do hypnotics increase the risk of driving accidents or near miss accidents due to hypovigilance? The effects of sex, chronic sleepiness, sleep habits and sleep pathology
Source: PLoS One. 2020 Jul 27;15(7):e0236404. doi: 10.1371/journal.pone.0236404 (PMC7384619; doi:10.1371/journal.pone.0236404)
Supplement: S1 File — (DOCX) [file pone.0236404.s002.docx]

**Questionnaire du sommeil**

- **Important : les champs marqués d'un astérisque rouge sont obligatoires.**
- **Pour commencer**
- Identifiant de votre médecin du sommeil :

Ce champ doit rester vide si votre médecin du sommeil ne vous a pas communiqué d’identifiant lors de votre demande de rendez-vous

- Vos troubles sont-ils réguliers (supérieur à 3 mois et plus de 3 fois par semaine) ? *

L'ensemble du questionnaire fait référence à des troubles qui vous gênent et qui sont fréquents. Répondre "Non" si les symptômes ne vous arrivent que très occasionnellement

- - Oui
  - Non
- Vos troubles apparaissent-ils par périodes ? *
  - Oui
  - Non
  - Ne Sait Pas
- Date de naissance : *


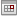


- Sexe*
  - Masculin
  - Féminin
- Poids (kg) : *

- Taille (cm) : *

- IMC

- Dans quel département habitez-vous ? (Sur 2 chiffres) *

0 de 2 caractères maximum

0 de 2 caractères maximum

- Vous vivez : *
  - Seul(e)
  - En couple
- Combien d'enfants vivent au foyer ? *

- **SYMPTOMES DES TROUBLES DU SOMMEIL**

**Ressentez-vous les symptômes suivants depuis plus de 3 mois, et avec des conséquences sur la qualité de votre journée ?**

- Des difficultés d’endormissement*
  - Oui
  - Non
- Des réveils en milieu de nuit*
  - Oui
  - Non
- Des réveils de fin de nuit /réveils précoces*
  - Oui
  - Non
- La sensation de ne pas dormir*
  - Oui
  - Non
- **Avez-vous des troubles respiratoires ?**
- Ronflez-vous ou avez-vous une respiration bruyante en dormant ? (Au moins 3 fois par semaine) *
  - Oui
  - Non
  - Ne Sait Pas
- Votre entourage a-t-il noté des arrêts respiratoires au cours du sommeil ? *
  - Oui
  - Non
  - Ne Sait Pas

**Êtes-vous somnolent ?**

**La somnolence n’est pas un simple ressenti de fatigue mais s’entend comme une lutte intense contre l’envie de dormir.**

- Avez-vous une somnolence excessive ou des accès d’endormissement involontaires (en dehors des siestes que vous organisez) tous les jours ou presque tous les jours ?
  - Oui
  - Non
  - Ne Sait Pas
- Faites-vous la sieste tous les jours ou presque tous les jours ? *
  - Oui
  - Non
- Etes-vous gêné par le besoin de lutter contre l’envie de dormir en cours de journée ? *
  - Oui
  - Non
- Avez-vous déjà eu un accident ou frôlé un accident au volant en cours de journée en raison de votre somnolence voire d’un endormissement involontaire, au cours des 6 derniers mois ? *
  - Oui
  - Non
- **Périodes de sommeil**
- Vos horaires de sommeil sont-ils très décalés par rapport aux horaires habituels de la vie sociale (notamment coucher et lever très tardifs ou coucher et lever très tôt) ?*
  - Oui
  - Non
- Spontanément vous ne dormez pas la nuit mais sur le matin et en début d'après-midi*
  - Oui
  - Non
- **Avez-vous d’autres troubles gênants et réguliers ?**
- Avez-vous des accès réguliers de somnambulisme (plusieurs fois par mois) ?*
  - Oui
  - Non
  - NSP
- Pendant le sommeil, avez-vous un comportement moteur anormal (ex : agitation nocturne, coups, cris…) plusieurs fois par mois ? *
  - Oui
  - Non
  - NSP
- Faites-vous fréquemment (plus d’une fois par semaine) des cauchemars et/ou des rêves pénibles ? *
  - Oui
  - Non
- **Au commencement**
- Savez-vous exactement quand ont débuté vos troubles ? *
  - Oui
  - Ne Sait Pas
- **EVALUATION DE LA SOMNOLENCE (QUESTIONNAIRE D’EPWORTH)**
- Voici quelques situations relativement usuelles, où nous vous demandons d’évaluer le risque de vous assoupir. Aussi, si vous n’avez pas été récemment dans l’une de ces situations, essayez d’imaginer comment cette situation pourrait vous affecter. Utilisez l'échelle suivante en cochant le chiffre le plus approprié pour chaque situation :
  - **0 = aucune chance de somnoler ou de s’endormir**
  - **1 = faible chance de s'endormir**
  - **2 = chance moyenne de s'endormir**
  - **3 = forte chance de s'endormir**
- Assis en train de lire*
  - 0
  - 1
  - 2
  - 3
- En train de regarder la télévision*
  - 0
  - 1
  - 2
  - 3
- Assis, inactif dans un lieu public (cinéma, théâtre, réunion) *
  - 0
  - 1
  - 2
  - 3
- Comme passager d'une voiture (ou transport en commun) roulant sans arrêt pendant une heure*
  - 0
  - 1
  - 2
  - 3
- Allongé l'après-midi lorsque les circonstances le permettent*
  - 0
  - 1
  - 2
  - 3
- Assis, en parlant avec quelqu'un*
  - 0
  - 1
  - 2
  - 3
- Assis au calme après un déjeuner sans alcool*
  - 0
  - 1
  - 2
  - 3
- Dans une voiture immobilisée depuis quelques minutes*
  - 0
  - 1
  - 2
  - 3
- **ÉVALUATION DE L’INTENSITE DES TROUBLES (ECHELLES GRADUEES DE 0 A 10)**
- Nous vous demandons de préciser ici comment vous percevez vos symptômes, en vous situant sur une échelle d’évaluation allant de 0 à 10.
- Intensité des troubles du sommeil : *

Choix allant de 0 (Ces troubles ne me gênent pas du tout) à 10 (Ces troubles me gênent beaucoup)

- - 0
  - 1
  - 2
  - 3
  - 4
  - 5
  - 6
  - 7
  - 8
  - 9
  - 10
- Qualité du sommeil effectif : *

Choix allant de 0 (Mon sommeil est mauvais) à 10 (Mon sommeil est excellent)

- - 0
  - 1
  - 2
  - 3
  - 4
  - 5
  - 6
  - 7
  - 8
  - 9
  - 10
- Qualité de l’éveil pendant la journée : *

Choix allant de 0 (Je suis somnolent(e) dans la journée) à 10 (Je suis bien éveillé(e) dans la journée)

- - 0
  - 1
  - 2
  - 3
  - 4
  - 5
  - 6
  - 7
  - 8
  - 9
  - 10

**SIGNES D’ORIENTATION ORGANIQUE**

- Avez-vous parfois l’impression d’étouffer en dormant ? *
  - Oui
  - Non
- Avez-vous des troubles de la sexualité / libido ? *
  - Oui
  - Non
- Vous levez-vous plus de 2 fois pour uriner en cours de nuit ? (Nycturie)*
  - Oui
  - Non
- Bougez-vous beaucoup en dormant ? *
  - Oui
  - Non
- Avez-vous, en vous couchant, des régurgitations acides dans le fond de la gorge ? (RGO)*
  - Oui
  - Non
- Avez-vous des sueurs nocturnes ? *
  - Oui
  - Non
- Ressentez-vous des maux de tête au réveil ? *
  - Oui
  - Non
- Etes-vous gêné par une toux nocturne ? *
  - Oui
  - Non
- Avez-vous des douleurs qui vous réveillent ou qui vous empêchent de dormir ? *
  - Oui
  - Non
- Avez-vous des problèmes de perte d’urine au cours du sommeil plusieurs fois par an ? *
  - Oui
  - Non
- Votre partenaire se plaint-il de recevoir des « coups de pied » tout au long de la nuit ? *
  - Oui
  - Non
  - Ne Sait Pas
- Avez-vous des sensations désagréables ou pénibles dans les jambes (et parfois les bras) qui vous gênent plus de 3 fois par semaine ? *
  - Oui
  - Non
- Vous arrive-t-il d’avoir une chute de la tête, d’un membre ou de tout votre corps, vous conduisant à des maladresses ou à des chutes ? *
  - Oui
  - Non
- Avez-vous parfois l’impression d’entendre, de voir ou de sentir des choses qui n’existent pas (comme dans un rêve ou un cauchemar éveillé), au moment du coucher ou en cas de fatigue dans la journée ? *
  - Oui
  - Non
- Avez-vous parfois l’impression d’être « paralysé » lorsque vous sortez de votre sommeil ou lorsque vous vous endormez ? *
  - Oui
  - Non
- Etes-vous obligé de prévoir plusieurs réveils ou alarmes pour arriver à vous lever le matin ? *
  - Oui
  - Non
- **SIGNES D’ORIENTATION PSYCHOLOGIQUE**
- Etes-vous anxieux ? *
  - Oui
  - Non
  - Ne Sait Pas
- Avez-vous connu une période dépressive ? *
  - Oui
  - Non
  - Ne Sait Pas
- Etes-vous actuellement en dépression ? *
  - Oui
  - Non
  - Ne Sait Pas
- **QUESTIONNAIRE HAD**

Pour répondre à ces questions, donnez la réponse qui exprime le mieux ce que vous avez éprouvé **au cours de la semaine qui vient de s’écouler.**

- 1 - Je me sens tendu(e) ou énervé(e) : *
  - La plupart du temps
  - Souvent
  - De temps en temps
  - Jamais
- 2 - Je prends plaisir aux mêmes choses qu'autrefois : *
  - Oui, tout autant qu'avant
  - Pas autant
  - Un peu seulement
  - Presque plus
- 3 - J'ai une sensation de peur comme si quelque chose d'horrible allait m'arriver : *
  - Oui, très nettement
  - Oui, mais ce n'est pas trop grave
  - Un peu, mais cela ne m'inquiète pas
  - Pas du tout
- 4 - Je ris facilement et vois le bon côté des choses : *
  - Autant que par le passé
  - Plus autant qu'avant
  - Vraiment moins qu'avant
  - Plus du tout
- 5 - Je me fais du souci : *
  - Très souvent
  - Assez souvent
  - Occasionnellement
  - Très occasionnellement
- 6 - Je suis de bonne humeur : *
  - Jamais
  - Rarement
  - Assez souvent
  - La plupart du temps
- 7 - Je peux rester tranquillement assis(e) à ne rien faire et me sentir décontracté(e) : *
  - Oui, quoi qu'il arrive
  - Oui, en général
  - Rarement
  - Jamais
- 8 - J'ai l'impression de fonctionner au ralenti : *
  - Presque toujours
  - Très souvent
  - Parfois
  - Jamais
- 9 - J'éprouve des sensations de peur et j'ai l'estomac noué : *
  - Jamais
  - Parfois
  - Assez souvent
  - Très souvent
- 10 - Je ne m'intéresse plus à mon apparence : *
  - Plus du tout
  - Je n'y accorde pas autant d'attention que je ne devrais
  - Il se peut que je n'y fasse plus autant attention
  - J'y prête autant d'attention que par le passé
- 11 - J'ai la bougeotte et n'arrive pas à tenir en place : *
  - Oui, c'est tout à fait le cas
  - Un peu
  - Pas tellement
  - Pas du tout
  - 12 - Je me réjouis à l'idée de faire certaines choses : *
  - Autant qu'avant
  - Un peu moins qu'avant
  - Bien moins qu'avant
  - Presque jamais
- 13 - J'éprouve des sensations soudaines de panique : *
  - Vraiment très souvent
  - Assez souvent
  - Pas très souvent
  - Jamais
- 14 - Je peux prendre plaisir à un bon livre ou à une bonne émission radio ou de télévision : *
  - Souvent
  - Parfois
  - Rarement
  - Très rarement

- **VOS HABITUDES**
- Êtes-vous fumeur (y compris la cigarette électronique avec nicotine) ? *
  - Oui
  - Non
- Buvez-vous régulièrement des boissons énergisantes ou excitantes (type cola, café…) ? *
  - Oui
  - Non
- Buvez-vous régulièrement des boissons alcoolisées (bière, vin, apéritifs…) ? *
  - Oui
  - Non
- Consommez-vous de la drogue ?*
  - Oui
  - Non
- Pratiquez-vous une activité sportive ?*
  - Oui
  - Non
- Avez-vous pris du poids récemment ?*
  - Oui
  - Non
- **VOS HORAIRES ET HABITUDES DE SOMMEIL**
- **Vers quelle heure vous couchez-vous en semaine ?**
- entre*

- et*

- **Vers quelle heure vous levez-vous en semaine ?**
- entre*

- et*

- **Vers quelle heure vous couchez-vous en période de repos/vacances ?**
- entre*

- et*

- **Vers quelle heure vous levez-vous en période de repos/vacances ?**
- Entre*

- Et*

- Une fois couché(e) que faites-vous le plus souvent ? (Plusieurs réponses possibles) *
  - Je cherche à dormir immédiatement
  - J’ai une activité sexuelle
  - Je lis un livre ou une revue
  - J’utilise mon téléphone/ ma tablette/ mon ordi pour échanger avec les autres (SMS, mails, réseaux sociaux) ou pour le travail
  - Je garde mon Smartphone allumé à côté de moi
  - Je regarde la télévision
  - Autre
- Il vous faut combien de temps pour vous endormir ? *

- Si vous vous réveillez en cours de nuit, durée des éveils ? *

- De combien d’heures de sommeil pensez-vous avoir besoin ? *

- **COMPORTEMENT**
- Sortez-vous le soir (soirée, théâtre, cinéma…) plus de 3 fois /semaine*
  - Oui
  - Non
- Juste avant le coucher, utilisez-vous régulièrement (plus de 3 fois/sem) un ordinateur ou une tablette pendant plus d’une heure ? *
  - Oui
  - Non
- Le soir, pesez-vous plus d’une heure dans votre lit à regarder votre téléphone, votre tablette ou votre ordinateur (plus de 3 fois/sem) ?*
  - Oui
  - Non
- En cours de nuit, répondez-vous à vos messages (mail, SMS, réseaux sociaux) ?*
  - Oui
  - Non
- Votre réveil est-il spontané sans alarme plus de 3 fois par semaine ?*
  - Oui
  - Non
- Comment vous sentez-vous le plus souvent au réveil ?*
  - en forme et dispos
  - encore fatigué et endormi
- Avez-vous une sensibilité de l’humeur à la lumière et au beau temps ?*
  - Oui
  - Non
  - Ne Sait Pas
- Travaillez-vous (professionnel ou domestique), le soir chez vous, jusqu’au coucher plus de 3 fois /sem ?*
  - Oui
  - Non
- **ÉTAT DE SANTE**
- Avez-vous de l’hypertension ?*
  - Oui
  - Non
- Avez-vous des troubles cardiaques ?*
  - Oui
  - Non
- Avez-vous du diabète ?*
  - Oui
  - Non
- Avez-vous des troubles respiratoires ?*
  - Oui
  - Non
- Avez-vous des troubles neurologiques ?*
  - Oui
  - Non
- Avez-vous des troubles psychiatriques ?*
  - Oui
  - Non
- Avez-vous des problèmes thyroïdiens ?*
  - Oui
  - Non
- Êtes-vous ménopausée ?*
  - Oui
  - Non
  - Non concerné
- Êtes-vous suivi pour une autre maladie ?*
  - Oui
  - Non
- **ANTECEDENTS FAMILIAUX**

Troubles du sommeil dont souffrent vos parents, frères et soeurs

- Syndrome d’apnée du sommeil ?*
  - Oui
  - Non
  - Ne sait pas
- Dépression ?*
  - Oui
  - Non
  - Ne sait pas
- Syndrome des jambes sans repos ?*
  - Oui
  - Non
  - Ne sait pas
- Narcolepsie*
  - Oui
  - Non
  - Ne sait pas
- Hypersomnie ?*
  - Oui
  - Non
  - Ne sait pas
- Autre ? Précisez :

- **TRAITEMENT(S) ACTUEL(S)**

Avez-vous un traitement pour :

- Une dépression ?*
  - Oui
  - Non
  - Ne Sait Pas
- L’anxiété ?*
  - Oui
  - Non
  - Ne Sait Pas
- Des douleurs ?*
  - Oui
  - Non
  - Ne Sait Pas
- Augmentation de la tension artérielle ?*
  - Oui
  - Non
  - Ne Sait Pas
- Du diabète ?*
  - Oui
  - Non
  - Ne Sait Pas
- Autre ?*
  - Oui
  - Non
  - Ne Sait Pas
- TRAITEMENT(S) MEDICAMENTEUX ACTUEL(S) :

Précisez si possible les médicaments utilisés. Si aucun, laissez vide.

- Prenez-vous des médicaments pour dormir ?*
  - Oui
  - Non
- Si ce questionnaire ne décrit pas exactement votre sommeil, merci de préciser :

- Merci de préciser votre catégorie socio-professionnelle ou activité :*

- Si vous êtes en activité :
- Précisez votre profession :

- Avez-vous des horaires de travail de nuit ?
  - Oui
  - Non
- Avez-vous des horaires de travail qui vous obligent à vous coucher très tard ou très tôt ?
  - Oui
  - Non
- Considérez-vous que votre travail soit stressant ?
  - Oui
  - Non

Enquête SPS sur le sommeil des soignants

**Si vous n’êtes pas professionnels de santé, validez dès maintenant votre questionnaire sans tenir compte des questions ci-dessous.**

- Etes-vous un professionnel de santé ?
  - Oui
  - Non
