# Supplementary material for: Do hypnotics increase the risk of driving accidents or near miss accidents due to hypovigilance? The effects of sex, chronic sleepiness, sleep habits and sleep pathology
Source: PLoS One. 2020 Jul 27;15(7):e0236404. doi: 10.1371/journal.pone.0236404 (PMC7384619; doi:10.1371/journal.pone.0236404)
Supplement: S2 File — (DOCX) [file pone.0236404.s003.docx]

Sleep questionnaire

*Please note that a participant cannot fill in the form if they do not consent to the conditions of use . All questions are mandatory, although certain questions only appear if the participant answers ‘yes’: for example the Insomnia Severity Index appears only if the participant reports symptoms of insomnia*

# To start

• Your sleep doctors identification number:

This field must remain empty if your sleep doctor did not give you an identifier when you requested an appointment.

Are your sleep problems regular (more than 3 months and more than 3 times a week)?

The entire questionnaire refers to troubles that bother you and that are frequent. Answer "No" if symptoms only happen to you very occasionally

• Yes

• No

• Do your disorders appear by periods?

• Yes

• No

Date of birth:

Sex

• Male

• Female

Weight (kg):

Height (cm):

BMI (calculated automatically)

In which department do you live ? (2 digits)

• Alone

• with someone

How many children live at home?

# SYMPTOMS OF SLEEP DISORDERS

Have you had the following symptoms for more than 3 months, and with negative effects on the quality of your day?

Difficulty falling asleep

• Yes

• No

Waking up in the middle of the night

• Yes

• No

Late night awakenings / early awakenings

• Yes

• No

The feeling of not sleeping

• Yes

• No

# Do you have respiratory problems?

Do you snore or breathe loudly while sleeping? (At least 3 times a week)

• Yes

• No

• Do not know

Do those around you notice your breathing stops during sleep?

• Yes

• No

• Do not know

# Are you sleepy?

*Drowsiness is not just a feeling of tiredness but means you have an intense fight against the urge to sleep.*

Do you have excessive drowsiness or involuntary fits of falling asleep (apart from organised) every day or almost every day?

• Yes

• No

• Do not know

Do you take a nap every day or almost every day?

• Yes

• No

Are you bothered by the need to fight the urge to sleep during the day?

• Yes

• No

Have you ever had an accident or had an accident while driving during the day because of your drowsiness or urge falling asleep in the past 6 months?

• Yes

• No

# Sleep periods

Are your sleep times very different from the usual hours of social life (especially going to bed and getting up very late or going to bed and getting up very early)?

• Yes

• No

Spontaneously you do not sleep at night but in the morning and early afternoon?

• Yes

• No

# Do you have other regular sleep problems?

Do you have regular sleepwalking attacks (several times a month)?

• Yes

• No

• Don’t know

During sleep, do you have abnormal motor behavior (e.g. agitation, blows, shouts, etc.) several times a month?

• Yes

• No

• Don’t know

Do you have frequent nightmares and / or painful dreams (more than once a week)?

• Yes

• No

# The start of your sleep problems

Do you know exactly when your troubles started?

• Yes

• Do not know

# SLEEPINESS ASSESSMENT (EPWORTH QUESTIONNAIRE)

Here are some relatively common situations where we ask you to assess the risk of falling asleep. Also, if you have not been in one of these situations recently, try to imagine how it could affect you. Use the following scale by checking the most appropriate number for each situation:

• 0 = no chance of dozing or falling asleep

• 1 = low chance of falling asleep

• 2 = average chance of falling asleep

• 3 = high chance of falling asleep

• Sitting reading

• Watching TV

• Sitting, inactive in a public place (cinema, theater, meeting)

• As a passenger in a car (or public transport) driving continuously for one hour

• Extended in the afternoon when circumstances allow

• Sitting, talking with someone

• Sitting in peace after an alcohol-free lunch

• In a car immobilized for a few minutes

# ASSESSMENT OF THE INTENSITY OF YOUR PROBLEMS (SCALES GRADUATED FROM 0 TO 10)

We ask you to specify here how you perceive your symptoms, by placing yourself on a rating scale going from 0 to 10.

- Intensity of sleep disorders: Choice going from 0 (These troubles do not bother me at all) to 10 (These troubles bother me a lot)
- Quality of sleep: Choice from 0 (My sleep is bad) to 10 (My sleep is excellent)
- Quality of wakefulness during the day: Choice from 0 (I am sleepy during the day) to 10 (I am well awake during the day)

# INSOMNIA SEVERITY INDEX

The Insomnia Severity Index has seven questions. The seven answers are added up to get a total score. When you have

your total score, look at the 'Guidelines for Scoring / Interpretation' below to see where your sleep difficulty fits.

For each question, please CIRCLE the number that best describes your answer.

Please rate the CURRENT (i.e. LAST 2 WEEKS) SEVERITY of your insomnia problem (s).

1. Difficulty falling asleep (None Mild Moderate Severe Very Severe)

2. Difficulty staying asleep (None Mild Moderate Severe Very Severe)

3. Problems waking up too early(None Mild Moderate Severe Very Severe)

4. How SATISFIED / DISSATISFIED are you with your CURRENT sleep pattern?

Very Satisfied Satisfied Moderately Satisfied Dissatisfied Very Dissatisfied

5. How NOTICEABLE to others do you think your sleep problem is in terms of impairing the quality of your life? ( not at all noticeable A little Somewhat Much Very much noticeable)

6. How WORRIED / DISTRESSED are you about your current sleep problem? ( Not at all worried A little Somewhat Much Very much worried)

7. To what extent do you consider your sleep problem to INTERFERE with your daily functioning (e.g. daytime fatigue, mood, ability to function at work / daily chores, concentration, memory, mood, etc.) CURRENTLY? ( Not at all interfering A little Somewhat Much Very much interfering)

# SYMPTOMS OF SLEEP PROBLEMS

Do you sometimes feel like you are suffocating while sleeping?

• Yes

• No

Do you have sexual / libido disorders?

• Yes

• No

Do you get up more than twice to urinate during the night? (Nocturia)

• Yes

• No

Do you move a lot while sleeping?

• Yes

• No

Do you have acid regurgitation in the back of your throat when you lie down? (GERD)

• Yes

• No

Do you have night sweats?

• Yes

• No

Do you experience headaches upon waking?

• Yes

• No

Are you bothered by a night cough?

• Yes

• No

Do you have pain that wakes you up or prevents you from sleeping?

• Yes

• No

Do you wet the bed while you are asleep several times a year?

• Yes

• No

Does your partner complain about being kicked all night long?

• Yes

• No

• Do not know

Do you have unpleasant or painful sensations in the legs (and sometimes the arms) that bother you more than 3 times a week?

• Yes

• No

If yes:

relieved by leg movements (stretching, walking)

- Yes
- No

occurring exclusively or mainly in the evening or at night

- Yes
- No

which start at rest or if you are inactive (lying down for example)?

- Yes
- No

Do you ever have a loss of muscle tone in your head, limb or entire body, leading to clumsiness or falls?

• Yes

• No

Do you sometimes have the impression of hearing, seeing or feeling things that do not exist (such as in a waking dream or nightmare), at bedtime or when you are tired during the day?

• Yes

• No

Do you sometimes feel like you are "paralyzed" when you get out of sleep or fall asleep?

• Yes

• No

Do you have to plan several alarm clocks or alarms to get up in the morning?

• Yes

• No

SYMPTOMS OF PSYCHOLOGICAL PROBLEMS

Are you anxious?

• Yes

• No

• Do not know

Have you experienced a period of depression?

• Yes

• No

• Do not know

Are you currently in depression?

• Yes

• No

• Do not know

# HAD QUESTIONNAIRE

To answer these questions, give the answer that best expresses what you have experienced in the past week.

1 I feel tense or 'wound up':

• Most of the time

• Often

• Sometimes

•Never

2 - I still enjoy the things I used to enjoy:

• Yes, just as much as before

•Not that much

•Just a little

• Almost more

3 - I get a sort of frightened feeling as if something awful is about to happen:

• Yes, very clearly

• Yes, but it's not too serious

• A little, but that doesn't worry me

• Not at all

4 - I can laugh and see the funny side of things

• As much as in the past

• Not as much as before

• Really less than before

•Not at all

5 - Worrying thoughts go through my mind:

• Very often

•Often enough

• Occasionally

• Very occasionally

6- I feel cheerful:

• Most of the time

• Often

• Sometimes

•Never

7- I can sit at ease and feel relaxed:

• As much as in the past

• Not as much as before

• Really less than before

•Not at all

8 - I feel as if I am slowed down:

• Most of the time

• Often

• Sometimes

•Never

9 -I get a sort of frightened feeling like 'butterflies' in the stomach:

• Very occasionally

• Occasionally

•Quite often

• Very often

10- I have lost interest in my appearance:

Not at all

• I don't pay as much attention to it as I should

• I may not be paying as much attention

• I pay as much attention to it as in the past

11 - I feel restless as I have to be on the move:

• Yes, this is absolutely the case

•A little

• Not really

• Not at all

• 12 - I look forward with enjoyment to things:

•As much as before

• A little less than before

• Much less than before

• Almost never

• 13 - I get sudden feelings of panic:

• Really very often

•Quite often

• Not very often

• Never

• 14 - I can enjoy a good book or a good radio or television program:

• Often

• Sometimes

• Rarely

•Very rarely

# YOUR HABITS

Are you a smoker (including electronic cigarettes with nicotine)?

• Yes

• No

Do you regularly drink energizing or caffeinated drinks (such as cola, coffee, etc.)?

• Yes

• No

Do you regularly drink alcoholic beverages (beer, wine, aperitifs, etc.)?

• Yes

• No

Do you use drugs?

• Yes

• No

Do you practice a sporting activity?

• Yes

• No

Have you gained weight recently?

• Yes

• No

# YOUR SLEEPING TIMES AND HABITS

Around what time do you go to bed during the week?

Around what time do you get up during the week?

Around what time do you go to bed during days off / vacation?

Around what time do you get up during the days off / vacation period?

Once in bed, what do you do most often?

• I want to sleep immediately

• I have sexual activity

• I read a book or a magazine

• I use my phone / tablet / computer to chat with others (SMS, emails, social networks) or for work

• I keep my Smartphone turned on next to me

• I watch TV

• Other

How long does it take you to fall asleep?

If you wake up during the night, how long are you awake?

How many hours of sleep do you think you need?

# SLEEP HABITS

Do you go out in the evening (evening, theater, cinema…) more than 3 times / week *

• Yes

• No

Just before bedtime, do you regularly (more than 3 times / week) use a computer or tablet for more than an hour?

• Yes

• No

In the evening, do you spend more than an hour in bed looking at your phone, tablet or computer (more than 3 times / week)?

• Yes

• No

During the night, do you respond to your messages (email, SMS, social networks)?

Yes

No

Do you wake up spontaneously without an alarm more than 3 times a week?

• Yes

• No

How do you feel most often upon waking?

• Wide awake

• still tired and asleep

• Other

Do you feel that you are positively affected by daylight and sunny weather?

• Yes

• No

• Do not know

Do you work (professional tasks or domestic chores), in the evening at home, until bedtime more than 3 times / week?

• Yes

• No

# YOUR HEALTH

Do you have high blood pressure ?

• Yes

• No

Do you have heart problems?

• Yes

• No

Do you have diabetes?

• Yes

• No

Do you have respiratory problems?

• Yes

• No

Do you have neurological problems?

• Yes

• No

Do you have psychiatric problems?

• Yes

• No

Do you have thyroid problems?

• Yes

• No

Are you menopausal?

• Yes

• No

• Not concerned

Are you being followed up for another illness?

• Yes

• No

# FAMILY HISTORY OF SLEEP DISORDERS

Do your parents or siblings suffer from

Sleep apnea syndrome ?

• Yes

• No

• Do not know

Depression?

• Yes

• No

• Do not know

Restless leg syndrome?

• Yes

• No

• Do not know

narcolepsy

• Yes

• No

• Do not know

Hypersomnia?

• Yes

• No

• Do not know

# YOUR CURRENT TREATMENT (S)

Do you have treatment for:

Depression ?

• Yes

• No

• Do not know

Anxiety?

• Yes

• No

• Do not know

Pain ?

• Yes

• No

• Do not know

Increased blood pressure?

• Yes

• No

• Do not know

Diabetes?

• Yes

• No

• Do not know

Other?

• Yes

• No

• Do not know

Do you take sleep medication?

• Yes

• No

With a medical prescription:

Zolpidem (Stilnox®) *

• Yes

• No

Zopiclone (Imovane®) *

• Yes

• No

melatonin *

• Yes

• No

Other, give details :

Without a medical prescription (over the counter at the pharmacy, internet ...):

Donormyl® *

• Yes

• No

Melatonin (alone or in combination) *

• Yes

• No

Phytotherapy (without associated melatonin) *

• Yes

• No

Other food supplements (without associated melatonin): magnesium, vitamins, amino acids ... *

• Yes

• No

# FURTHER INFORMATION

If this questionnaire does not exactly describe your sleep, please give details:

Please specify your socio-professional category or activity:

If you work:

Specify your profession:

Do you do night work schedules?

• Yes

• No

Do you have working hours that require you to go to bed very late or very early?

• Yes

• No

Do you consider your work to be stressful?

• Yes

• No
